# How to divide the pancreatic parenchyma in patients with a portal annular pancreas: laparoscopic spleen-preserving distal pancreatectomy for serous cystic neoplasms

**DOI:** 10.1186/s40792-020-00852-7

**Published:** 2020-05-01

**Authors:** Naohisa Kuriyama, Tomohide Hatanaka, Kazuaki Gyoten, Aoi Hayasaki, Takehiro Fujii, Yusuke Iizawa, Hiroyuki Kato, Yasuhiro Murata, Akihiro Tanemura, Masashi Kishiwada, Hiroyuki Sakurai, Shugo Mizuno

**Affiliations:** Department of Hepatobiliary Pancreatic and Transplant Surgery, Mie University Graduate School of Medicine, 2-174 Edobashi, Tsu City, 514-8507 Mie Japan

**Keywords:** Portal annular pancreas, Spleen-preserving distal pancreatectomy, Laparoscopic surgery, Postoperative pancreatic fistula

## Abstract

**Background:**

Portal annular pancreas (PAP) is a rare pancreatic anomaly in which the uncinate process wraps annularly around the portal vein and fuses to the body of the pancreas. PAP is highly relevant to the development of postoperative pancreatic fistula (POPF) in pancreatic surgery. Here, we describe our experience and surgical technique of laparoscopic spleen-preserving distal pancreatectomy using Warshaw’s procedure for patients with the PAP.

**Case presentation:**

A 49-year-old woman with PAP was referred to our hospital for a suspicious mucinous cystic neoplasms 1.5 cm in diameter in the pancreatic tail. Laparoscopic spleen-preserving distal pancreatectomy using Warshaw’s procedure was performed. Mobilization of the pancreatic tail was first performed, and then, the splenic artery was cut. After dividing the pancreatic tail from the splenic hilum, the ventral pancreatic parenchyma was divided using a stapler. After cutting the splenic vein, complete mobilization of the pancreatic body and tail enabled easy division of the PAP. Finally, the PAP was also divided using the stapler. Although grade B POPF occurred, she was discharged on the 9th postoperative day.

**Conclusions:**

Surgeons should understand the anatomical characteristics of PAP and be aware of the possibility of POPF.

## Background

Portal annular pancreas (PAP) or the so-called circumportal pancreas is a rare pancreatic anomaly without symptoms, in which the uncinate process of the pancreas encircles the portal vein (PV) and/or its influx, the superior mesenteric vein (SMV), and the splenic vein (SV), and extends to the dorsal surface of the pancreas body [1]. In 1987, Sugiura et al. first reported PAP as the hypertrophic uncinate process surrounding the superior mesenteric artery (SMA) and SMV [2].

Here, we describe a case of a patient with a serous cystic neoplasm with the PAP who underwent laparoscopic spleen-preserving distal pancreatectomy (DP) using Warshaw’s procedure.

## Case presentation

**Fig. 1:**
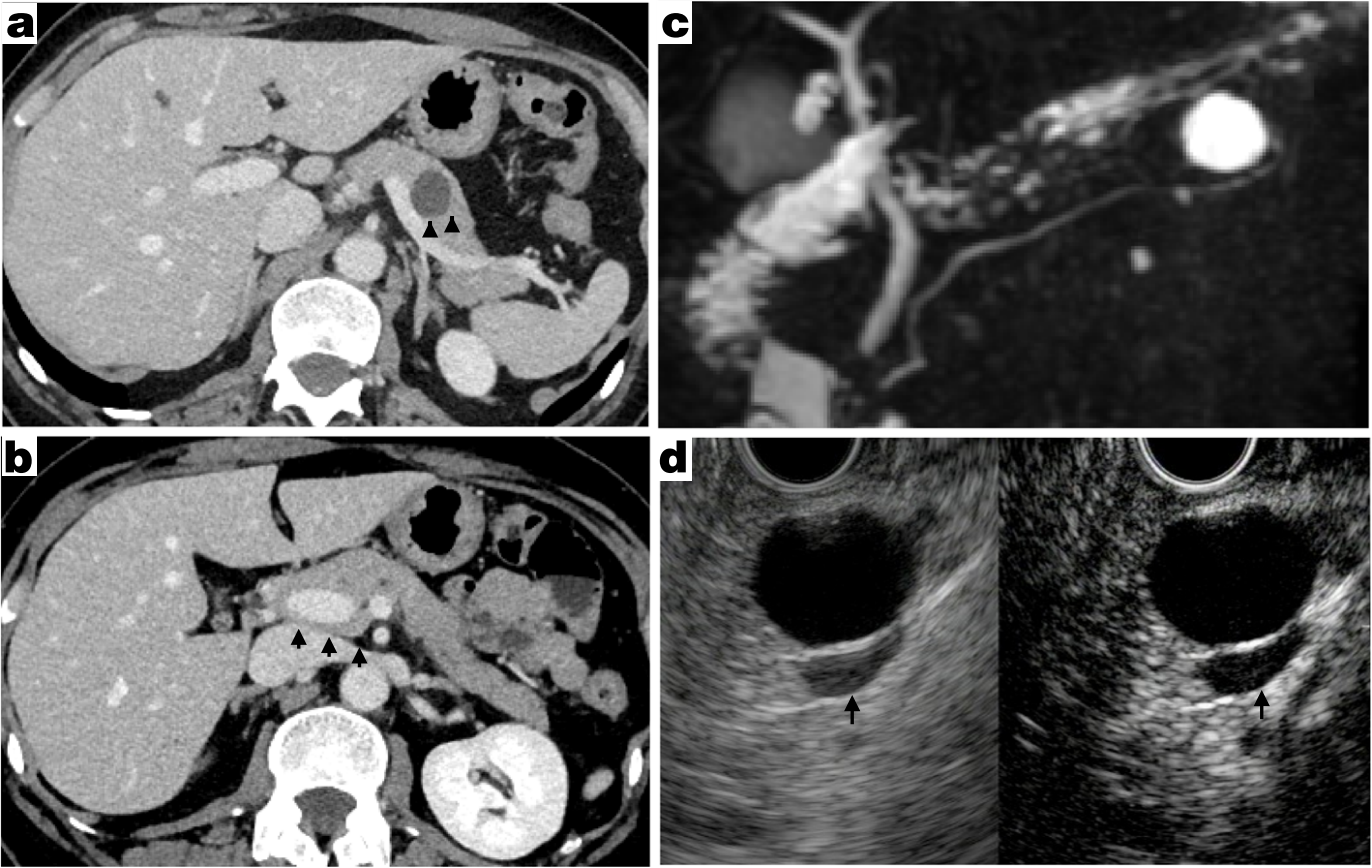
Preoperative imaging studies. Dynamic computed tomography scan showed oval cystic lesion of 1.5 cm in diameter of the pancreatic tail (**a**: arrow head) and portal annular pancreas (**b**: arrow). **c** Magnetic resonance cholangiopancreatography demonstrated the anteportal main pancreatic duct type. **d** Contrast-enhanced endoscopic ultrasound revealed oval cystic lesion without enhancement

A 47-year-old female (body mass index, 20.5) had an oval cystic lesion in the pancreatic tail identified by an annual health check and then visited the nearby hospital for further examination. She had a medical history of a left parapelvic cyst at 44 years old. Plain computed tomography (CT) scans taken at the same hospital 4 years ago did not indicate any pancreatic cysts. She was referred to our hospital for the surgical treatment of a suspected mucinous cystic neoplasm (MCN) without a mural nodule that was 1.5 cm in diameter in the pancreatic tail (Fig. 1a). A preoperative dynamic CT scan showed that the PV was annularly surrounded by pancreatic parenchyma at the superior side of the SV (Fig. 1b). Magnetic resonance cholangiopancreatography demonstrated the anteportal main pancreatic duct (MPD) (Fig. 1c). Contrast-enhanced endoscopic ultrasound revealed an oval cystic lesion without enhancement (Fig. 1d). The preoperative imaging studies revealed the PAP classified as type IIIA according to the Karasaki and Joseph classification [3, 4]. Finally, the patient was diagnosed with a suspected MCN with PAP, and laparoscopic spleen-preserving DP using Warshaw’s procedure was planned. Preoperative laboratory data is shown in Table 1, and carbohydrate antigen 19-9 levels was slightly elevated.

**Table 1:** Preoperative laboratory data

TP	7.0 g/dL	WBC	4300/uL
Alb	4.3 g/dL	RBC	/uL
AST	17 U/L	Hb	13.4 g/dL
ALT	10 U/L	Ht	40.1%
T-Bil	0.6 mg/dL	PLT	25.1/uL
D-Bil	0.1 mg/dL	Tumor marker	
ALP	196 U/L	CEA	1.3 ng/mL
γGLP	15 U/L	CA19-9	45.2 U/mL
BUN	13 mg/dL	(normal rage : less than 36.8 U/mL)	
Cr	0.58 mg/dL		
Amy	154 U/L		
Glu	105 mg/dL		
HbA1c	5.4%		
CRP	0.05 mg/dL		

*CEA* carcinoembryonic antigen, *CA19-9* carbohydrate antigen 19-9

**Fig. 2:**
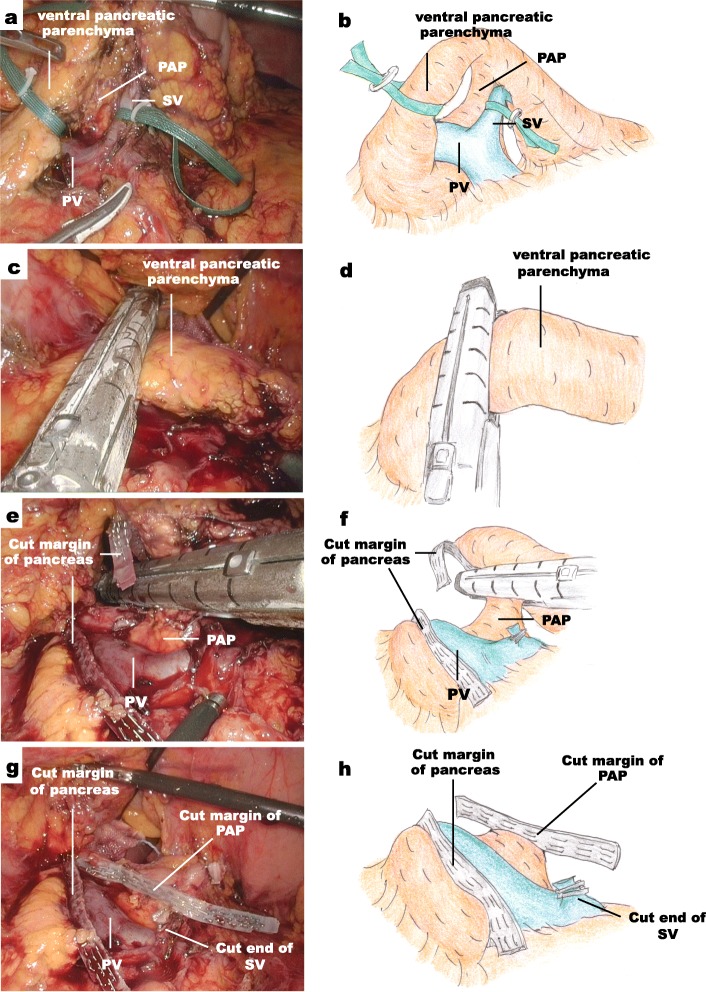
Intraoperative findings. **a**, **b** Exposure of the PAP after taping the pancreatic body and SV. **c**, **b** Dividing of ventral pancreatic parenchyma using stapler after dividing the pancreatic body. **e**, **f** Dividing of PAP using the stapler. **g**, **h** Operative field after dividing the PAP. PAP, portal annular pancreas; PV, portal vein; SV, splenic vein

Under general anesthesia, the patient was placed in the lithotomy position. Five ports were inserted in the following order: (a) a 12-mm port inserted through the umbilicus to introduce the laparoscope by the open method, (b) a 12-mm port at the right lateral side of the umbilicus on the midclavicular line, (c) a 5-mm port in the right hypochondriac region on the midclavicular line, (d) a 12-mm port at the left lateral side of the umbilicus on the midclavicular line, and (e) a 5-mm port in the left hypochondriac region on the anterior axillary line. After the bursa omentalis was widely opened toward the border of the preserved left gastroepiploic vessels that ultimately fed the spleen, the splenocolic ligament was divided to take down the splenic flexure of the transverse colon. The serosa of the inferior border of the pancreatic body and tail was cut, and then, the pancreatic tail was widely mobilized from the retroperitoneum. After the greater curvature of the stomach was suspended with two sutures using a 2-0 nylon thread to expose the pancreas, the serosa of the superior border of the pancreas was cut, and then, the common hepatic artery and splenic artery (SA) were exposed. At this time, the cranial wall of the PAP at the left side of the PV was identified. The SA was occluded at its root using Hem-o-lok clips and then was divided to block inflow to spleen. The pancreatic tail was completely mobilized from the retroperitoneum. After exposing the splenic hilum, some branches of the SV and SA were carefully clipped and cut at the end of the distal pancreas without injury to the left gastroepiploic and short gastric vessels. Finally, the ventral pancreatic parenchyma was tapped through the ventral side of the PV/SMV (Fig. 2a) and then was divided using the Endo GIA™ reinforced with a Tri-Staple™ reload (purple reload, 60 mm, Fig. 2b). After dividing the SV at its root, the dorsal side of the PAP was finally divided using the previously mentioned stapler (Fig. 2c, d), and then, the resected specimen was extracted using a retrieval bag. This operative time was 268 min, and blood loss was 175 mL.

During postoperative course, the grade B postoperative pancreatic fistula (POPF) occurred, and the antibiotics treatment alone was done. She was discharged on the 9th postoperative day and was pathologically diagnosed with serous cystic neoplasm.

## Discussion

PAP is a rare congenital pancreatic anomaly present in 0.5 to 2.4% of normal people [1, 5–7]. In 2009, Karasaki et al. reported three classification types of PAP (A: suprasplenic, B: infrasplenic, and C: mixed type), depending on the relation to the portal confluence. The most frequent in the literature is type A, which ranges in incidence between 70 and 94%, followed by type B with an incidence between 4 and 20%, and type C with an incidence between 2 and 14% [1, 3, 5]. In 2010, Joseph et al. suggested three types of PAP (I: retroportal, II: type I is associated with pancreas divisum, and III: anteportal) based on the course of the MPD and then classified type III into three subtypes (IIIA, IIIB, and IIIC) according to the Karasaki classification. Type IIIC was the most frequent type, with an incidence that ranges between 44 and 92% [1, 5]. Yilmaz and Ahmed also reviewed 55 cases of PAP and reported vascular variations in 16 (29%) cases. Among them, most were treated by replacing the right hepatic artery with the superior mesenteric artery (*n* = 6). The authors concluded that identifying the PAP and defining the pancreatic duct and vascular variations are important to preventing possible complications in patients undergoing pancreatic surgery.

In terms of the pancreatic division procedure for patients with PAP who undergo DP, there are two procedures: one cut margin or two cut margins. Ohtsuka et al. discussed the two procedures in detail: pancreatic division at the level of the PV/SMV, yielding two cut margins of the pancreatic parenchyma, and pancreatic division on the distal side of the PAP, resulting in a single cut margin. They recommended that two cut margins must be made during DP at the level of the PV/SMV because a single cut margin usually leads to a larger area of the cut surface in the pancreas body than at the level of the PV/SMV. In the case of a one cut margin, the line of pancreatic division is the distal side of the PAP, as Ohtsuka et al. mentioned [7]. In this case, the thickness of the pancreas at the level of the SMA was 12 mm, which was larger than the 9 mm on the ventral side of the SMV. The distance between the distal side of the PAP and the right edge of the tumor was 12 mm, which is not enough distance to perform pancreatic division using a surgical stapler with a one cut margin. Thus, we selected two cut margins to secure the surgical margin and to prevent POPF. The rate of POPF depends on the thickness of the pancreatic parenchyma at the cut line as well as the number of cut surfaces of the pancreas (one cut margin/two cut margin). In previous papers, a pancreatic transection line thickness of 12 mm or larger was a risk factor for POPF [8, 9]. Therefore, the one margin procedure is better for PAP patients as long as the thickness of the pancreatic parenchyma at the cut line is less than 12 mm; otherwise, a two cut margin procedure should be recommended. The cystic tumor of the pancreatic tail in this case was in complete contact with the splenic vein. Laparoscopic spleen-preserving distal pancreatectomy using Warshaw’s procedure was planned because we assumed it was impossible to detach this tumor from the splenic vein without exposing the cystic wall. Recently, Song et al. reported that distal pancreatectomy using the splenic vessel-preserving technique led to a significantly lower incidence of clinically relevant postoperative pancreatic fistula, splenic infarcts, intra- and postoperative splenectomies, and gastric varices than that using Warshaw’s procedure [10]. Benign or borderline malignant tumors of the pancreatic tail completely separated from the SA and SV are considered good indications for distal pancreatectomy using the splenic vessel-preserving technique. To the best of our knowledge, 7 patients with PAP (type IA in 2, and type IIIA in 5) who underwent DP for several kinds of pancreatic disease have been reported in the English-language literature [1, 7, 11–13] including this case (Table 2). All patients had two pancreatic cut margins. Among them, 5 patients (71%) developed POPF (grade A in 2, and grade B in 3). Therefore, DP for patients with PAP led to a substantially increased risk for POPF as well as for pancreaticoduodenectomy [1]. In 2012, Jang et al. first reported laparoscopic DP for intraducta1 papillary mucinous neoplasm patients with PAP. However, the details of the operative procedure are unknown. In our case, the ventral pancreatic parenchyma was first divided. After cutting the SV, the complete mobilization of the pancreatic body and tail enabled easy division of the remnant PAP. It was considered that our surgical procedure was rational and useful to preventing PAP injury.

**Table 2:** Reported 7 cases with portal annular pancreas who underwent distal pancreatectomy

Author	Year	Age	Gender	Pancreatic disease	Classification of PAP	Laparoscopic surgery	No. of cut margin	POPF	Postoperative hospital stay
Hashimoto et al. [13]	2009	39	Female	MCN	Ia	No	2	Yes (grade A)	9
Jang et al. [11]	2012	74	Female	IPMN	IIIa	**Yes**^**a**^	2	Yes (grade A)	16
Yamaguchi [12]	2013	80	Female	Pancreas sarcoidosis	Ia	No	2	No	–
Harnoss et al [1]	2014	48	Female	Suprarenal cancer	IIIa	No	2	Yes (grade B)	13
Ohtsuka et al. [7]	2016	63	Male	PNET	IIIa	No	2	No	–
Ohtsuka et al. [7]	2016	61	Female	PDAC	IIIa	No	2	Yes (grade B)	–
Our case	2020	47	Female	SCN	IIIa	**Yes**	2	Yes (grade B)	9

*PAP* portal annular pancreas, *POPF* postoperative pancreatic fistula, *MCN* mucinous cystic neoplasm, *IPMN* intraductal papillary mucinous neoplasm, *PNET* pancreatic neuroendocrine tumor, *PDAC* pancreatic ductal adenocarcinoma, *SCN* serous cystic neoplasm

^a^Details of the surgical procedure were unknown

## Conclusion

It was considered that laparoscopic DP for patients with PAP was feasible. However, surgeons should understand the anatomical characteristics of PAP and be aware of the possibility of POPF.

## Data Availability

There is no available data and materials to be shared.

## Abbreviations

CT: Computed tomography
DP: Distal pancreatectomy
MCN: Mucinous cystic neoplasm
MPD: Main pancreatic duct
PAP: Portal annular pancreas
POPF: Postoperative pancreatic fistula
PV: Portal vein
SA: Splenic artery
SMA: Superior mesenteric vein
SMV: Superior mesenteric vein
SV: Splenic vein
